# Fibrinogen-to-Albumin Ratio as Predictor of Mortality in Acute Aortic Syndromes

**DOI:** 10.3390/jcm14051669

**Published:** 2025-03-01

**Authors:** Alexandra Julia Lipa, Patrick Andreikovits, Marco Stoeckl, Hans Domanovits, Christian Schoergenhofer, Michael Schwameis, Juergen Grafeneder

**Affiliations:** 1 Department of Emergency Medicine, Medical University of Vienna, 1090 Vienna, Austriajuergen.grafeneder@meduniwien.ac.at (J.G.); 2 Department of Clinical Pharmacology, Medical University of Vienna, 1090 Vienna, Austria

**Keywords:** emergency medicine, acute aortic syndrome, cardiovascular disease, biomarkers

## Abstract

**Background:** Acute aortic syndrome (AAS) is a life-threatening condition characterized by a high mortality, yet reliable prognostic biomarkers are still lacking. The fibrinogen-to-albumin ratio (FAR) has recently gained attention in cardiovascular research but has not been explored in the context of AAS. This study assessed the association between the FAR and 30-day mortality in AAS patients who presented to the emergency department. **Methods:** We included all AAS patients aged 18 years and older who presented to the emergency department between 2013 and 2021. The outcome measured was 30-day all-cause mortality. Cox regression analysis assessed the relationship between the FAR and the outcome. **Results:** A total of 171 patients (mean age 67, SD 13.7; 33% female) were included, with 68 (40%) dying within 30 days of admission. Non-survivors had a significantly lower FAR (mean 8.9, SD 4.97) than survivors (mean 10.8, SD 5.44, *p* = 0.02). FAR was significantly associated with 30-day mortality (crude HR 0.935, 95% CI 0.88–0.99, *p* = 0.02). This association remained significant after adjusting for age, sex, cardiopulmonary resuscitation, catecholamine administration, bleeding on admission, and type of AAS (adjusted HR 0.92, 95% CI 0.87–0.98, *p* = 0.01). **Conclusions:** FAR was independently associated with 30-day mortality in AAS patients who presented to the emergency department. Given its simplicity and cost-effectiveness, it could be a valuable biomarker, especially in resource-limited settings, to improve risk assessment and optimize resource allocation in AAS.

## 1. Introduction

Acute aortic syndrome (AAS) includes various aortic pathologies, mainly intramural hematoma (IMH), penetrating aortic ulcer (PAU), and acute aortic dissection (AAD) [1,2,3]. The annual incidence of AAS ranges from 2.9 to 6 per 100,000 patients [4,5]. Its highly variable clinical presentation, often mimicking other conditions, complicates rapid diagnosis. Its symptoms vary depending on the affected aortic segment, contributing to delays in recognition [6]. A longitudinal study revealed that only 15% of aortic dissection cases were diagnosed at the initial presentation, while 85% experienced delayed management.

### 1.1. Biomarkers in AAS

D-dimer is the most sensitive and widely used biomarker for AAS, particularly AAD [7]. As a degradation product of fibrinolysis [8], it is commonly used to rule out pulmonary embolism and AAD in clinical practice [9,10]. However, normal D-dimer levels have been reported in patients with IMH and PAU, highlighting the need for additional diagnostic biomarkers [5,11,12]. Other biomarkers, including inflammatory markers, tumor markers (such as tumor necrosis factor α and interleukin 8) [13], and smooth muscle markers (smooth muscle myosin heavy chains, creatinine kinase-BB isozyme, and calponin), have shown potential correlations with AAS disease severity and treatment [11,13] but have yet to be integrated into current diagnostic pathways [10,14].

### 1.2. Fibrinogen-to-Albumin Ratio

The fibrinogen-to-albumin ratio (FAR) has recently moved to the center of attention as a diagnostic and prognostic parameter for several cardiovascular diseases. It integrates the levels of fibrinogen and albumin, linking coagulation, inflammation, and hemodynamic changes in the body [15].

Both parameters are part of routine laboratory investigations and, therefore, are ubiquitous and cost-effective. Additionally, the ratio is simple to calculate and can be applied in various clinical settings. It has been extensively studied in oncology, with a recent meta-analysis demonstrating a significant association between the FAR and overall survival in cancer patients [16].

In neuro-oncological patients with glioblastoma, an elevated FAR demonstrated a stronger association with mortality than either fibrinogen or albumin alone [17]. The FAR may indicate systemic inflammation, and an elevated FAR has been observed in spondylarthritis [18] and systemic lupus erythematosus [19].

In myocardial infarction, the FAR independently predicts short-term mortality in patients undergoing percutaneous coronary intervention [20]. In ST-segment elevation myocardial infarction, significantly higher periprocedural FAR values were observed in patients who died [21]. An increased FAR has also been associated with higher rates of left ventricular systolic dysfunction in acute coronary syndrome [22].

Fibrinogen is a crucial protein in the coagulation process. In AAD, fibrinogen levels can be significantly reduced, likely due to consumption coagulopathy, which has been associated with worse outcomes, including an increased mortality [23]. Similarly, hypoalbuminemia has been associated with worse outcomes in patients with AAD [24], possibly reflecting inflammation, tissue damage, and vascular leakage. Although both biomarkers have been studied individually, the prognostic value of the FAR in AAS has yet to be explored.

## 2. Materials and Methods

### 2.1. Setting

This retrospective study included all patients aged 18 years and older with AAS who presented to the Department of Emergency Medicine at the Medical University of Vienna, situated within the Vienna General Hospital, from 2013 to 2021. The study received approval from the Ethics Committee of the Medical University of Vienna (EK Nr 1274/2021, approval date on 29 April 2021) and adhered to the Declaration of Helsinki. Vienna General Hospital, a 1500-bed tertiary care center, treats approximately 80,000 patients annually, with an average of 40 patients diagnosed with AAS. The department of Emergency Medicine includes both an outpatient ward and an intensive care unit. Blood samples were collected upon admission to the emergency department.

### 2.2. Inclusion and Exclusion

We included patients 18 years of age and older with AAS confirmed by radiologic imaging. We excluded patients with recurrent presentations or missing laboratory values for the FAR calculation.

### 2.3. Statistical Analysis

Baseline characteristics were analyzed using the mean and standard deviation (SD) or the median and interquartile range (IQR), as appropriate. We assessed data distribution with histograms and the Shapiro–Wilk test. For categorical variables, we calculated absolute and relative frequencies. We conducted between-group testing for categorical variables using the chi-squared test or Fisher’s exact test. The Mann–Whitney U test or *t*-test was applied for continuous variables. We did not conduct a formal sample size calculation for this retrospective observational study analyzing patient data from 2013 to 2021. The availability of eligible cases during this period determined the sample size.

We calculated the FAR by dividing fibrinogen levels (g/L, reference level 2–4 g/L) by albumin levels (g/L, reference level 35–52 g/L) and multiplying the results by 100.

The primary endpoint was 30-day all-cause mortality, and Cox regression was employed to explore its crude association with the FAR. In the second step, the following covariates were incorporated into the Cox regression: age, sex, cardiopulmonary resuscitation (CPR), catecholamine administration, and bleeding at admission. The results are presented as hazard ratios (HRs) with 95% confidence intervals (CIs). We used Schoenfeld residuals to assess the proportional hazards assumption and the Variance Inflation Factor to evaluate multicollinearity. Kaplan–Meier analysis (log-rank test) was performed to verify the time-dependent discriminative power of the calculated FAR quartiles.

Missing data were noted as such, and no imputation was conducted. A two-sided *p*-value of <0.05 was deemed statistically significant. Analyses were conducted using IBM SPSS Statistics Version 27.0.1, R (R Foundation for Statistical Computing, Vienna, Austria, http://www.R-project.org (accessed on 5 February 2025), version 3.6.2), and Microsoft Excel.

## 3. Results

**Figure 1:**
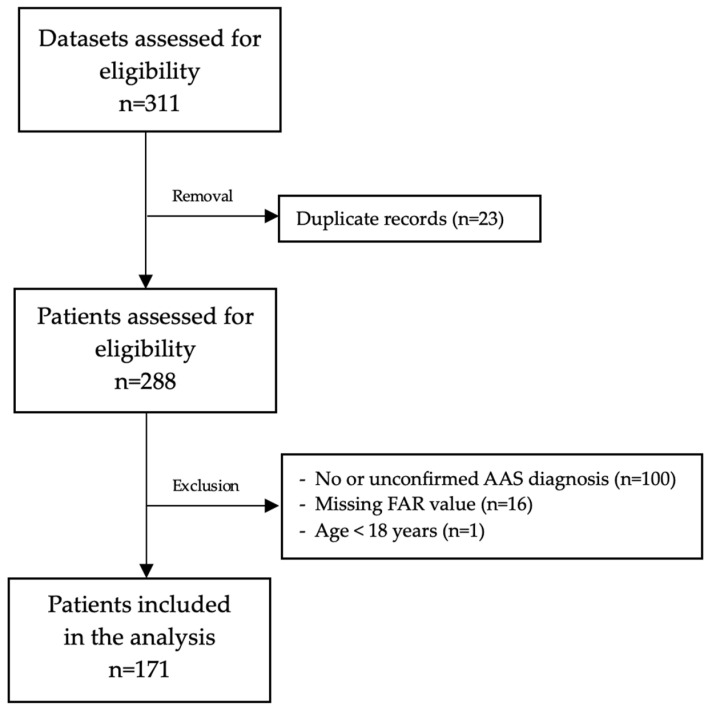
Flow diagram.

Figure 1 illustrates the inclusion and exclusion process for patients. Initially, 311 datasets were evaluated for eligibility. 171 patients were finally included in the analysis. Of these, 56 (33%) were female. The mean age was 67 (SD 13.7) years. The baseline characteristics of the study patients, categorized by 30-day mortality, are presented in Table 1. Overall, 68 patients (40%) died within 30 days of admission. Approximately half of these patients (n = 29) died on the day of admission. Patients who died within 30 days had higher rates of catecholamine administration (44% vs. 79%, *p* < 0.001), greater bleeding rates upon admission (80% vs. 94%, *p* = 0.009), and more frequent CPR (5% vs. 44%, *p* < 0.001).

Most patients (n = 107, 62.6%) were diagnosed with aortic dissection. Thirty-seven cases (21.6%) had aortic rupture, while four patients (2.3%) had ruptured aortic dissection. Aortic aneurysms were observed in 23 cases (13.5%). Additionally, four patients (2.3%) were diagnosed with an IMH, and five patients (2.9%) had a PAU. Notably, one patient (0.6%) experienced both an IMH and PAU.

Survivors had a higher FAR than non-survivors (*p* = 0.025, Table 2). FAR was significantly associated with 30-day mortality (crude HR 0.935, 95% CI: 0.88–0.99, *p* = 0.020). This association remained significant when the covariates sex, age, CPR, catecholamine administration, bleeding on admission, and type of AAS were added to the model (adjusted HR 0.92; *p* = 0.01). The respective HRs with their corresponding 95% CIs are shown in Table 3.

**Figure 2:**
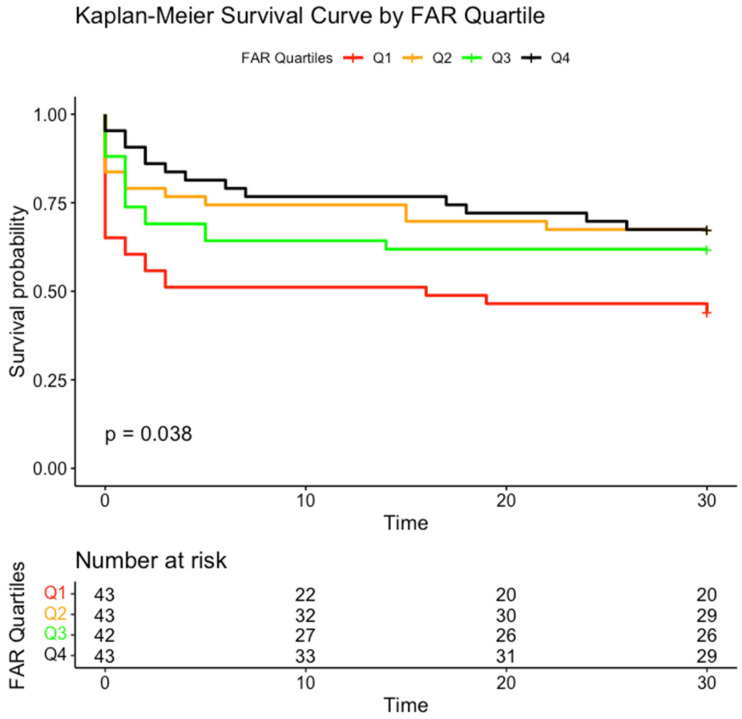
Kaplan–Meier curve categorized by fibrinogen-to-albumin ratio (FAR) quartiles.

Figure 2 displays the Kaplan–Meier survival curves categorized by FAR quartiles, illustrating the relationship between the FAR and 30-day survival. The survival analysis demonstrates a statistically significant difference among the quartiles (*p* = 0.038), with Quartile 1 exhibiting the lowest survival rate.

D-dimer levels were present in only 45 cases (26.3%). A significant correlation was found between D-dimer and FAR (r = −0.486, *p*-value = 0.001). We were able to calculate the 2018 International Society on Thrombosis and Haemostasis (ISTH) Disseminated Intravascular Coagulation (DIC) score [25,26] in 37 patients (Table 4). Among these 37 patients, 3 had overt DIC.

## 4. Discussion

This study is the first to demonstrate a significant association between the FAR and 30-day mortality in AAS patients. We observed an inverse relationship, where patients who died had a lower FAR (mean: 8.89) compared to survivors (mean: 10.74).

The average fibrinogen level was lower in non-survivors than in survivors (2.76 g/L vs. 3.75 g/L), possibly due to consumption, as indicated by the lower platelet count (169 G/L vs. 224 G/L) and higher D-dimer levels (63.8 vs. 8.1 µg/mL) observed in non-survivors. However, D-dimer levels were available for only a minority of patients (n = 45), which limits the strength of this conclusion. D-dimer levels in AAS can be seen as an indicator of secondary fibrinolysis. It is often used to rule out AAS and has also been shown to be useful regardless of symptom onset [27,28]. These findings suggest that D-dimer levels might correlate with disease severity rather than timeframe [28]. However, studies have indicated that, in cases of AAS with a thrombosed false lumen, including IMH and PAU, D-dimer levels may be negative, which limits accuracy in these cases [29,30,31]. The FAR and D-dimer levels demonstrated a significant negative correlation, supporting the assumption that a low FAR may be associated with fibrinolysis. Fibrinolysis results in elevated D-dimer levels and decreased fibrinogen levels, leading to a lower FAR.

Inflammation can contribute to reduced albumin levels. However, considering the timing of blood sampling relative to the disease stage, elevated inflammatory markers may not be detectable yet. In our data, C-reactive protein (CRP) levels and white blood cell counts did not differ significantly between those who survived and those who did not (CRP: 0.89 mg/dL vs. 0.66 mg/dL; white blood cell count: 12.4 G/L vs. 12.5 G/L). These findings suggest that the lower albumin levels observed may be attributed to fluid resuscitation rather than inflammation. Low fibrinogen levels due to consumption [32] may primarily account for the lower FAR in non-survivors. It is important to note that volume resuscitation may lead to hemodilution and decreased albumin and fibrinogen levels. However, the FAR remains unchanged, as both parameters are diluted equally.

As mentioned, decreased fibrinogen levels in AAS are primarily framed in the context of consumption coagulopathy and a concomitant increase in fibrinolysis [33]. While we observed normal fibrinogen levels in this study, significantly lower levels were observed in non-survivors. A possible explanation for the normal fibrinogen levels could be the very early blood draw at ED presentation. Findings that support the results of this study indicate that serum fibrinogen levels below 4 g/L are associated with increased mortality in Type-A aortic dissection [34]. Considering the data from this study, both survivors and non-survivors had mean serum fibrinogen levels below that cut-off, highlighting the extent of coagulation activation and consumption in AAS.

Several risk stratification tools are available for AAS, including the Aortic Dissection Detection Risk Score (ADD-RS) [35]. However, the ADD-RS is limited to identifying aortic dissections, overlooking approximately one-third of AAS patients in our cohort. Additionally, it relies on a comprehensive medical history, which may be challenging to obtain in emergencies (e.g., patients who are intubated or unconscious). In contrast, the FAR is entirely biomarker-based, making it accessible for nearly all patients and reflecting systemic inflammation and coagulation disturbances that are critical in AAS. Future studies should explore whether incorporating the FAR into risk models like the ADD-RS could enhance predictive accuracy and improve clinical decision making.

Interestingly, our findings differ from prior research investigating the FAR in cardiovascular disease. Zhao et al. [20] analyzed 510 patients with ST-segment elevation myocardial infarction. They reported contrasting results—a higher FAR was linked to increased 30-day mortality and a greater occurrence of no-reflow in the coronary artery. A FAR cut-off of 10.89 was identified in this study, with a higher FAR associated with worse outcomes. The authors suggest that this relationship may stem from fibrinolysis driven by low albumin levels and elevated fibrinogen as indicators of chronic inflammation in coronary artery disease. Despite the significant association found in their study, Zhao et al. deemed the utility of the FAR in acute coronary syndrome limited [20]. In our AAS cohort, the survivors had a mean FAR of 10.74. In contrast, non-survivors had a mean of 8.89. Similarly, Wang et al. [22] studied 650 patients with acute coronary syndromes. They discovered that patients with left ventricular systolic dysfunction exhibited a higher FAR than those with normal left ventricular systolic function (91.37 vs. 75.14, *p* < 0. 001). The authors hypothesized that the FAR might correlate more strongly with chronic inflammation than with albumin or fibrinogen levels. With this link to inflammation, the FAR may indicate remodeling processes contributing to left ventricular dysfunction in acute coronary syndrome [22]. These discrepancies could result from the differences between chronic and sub-acute diseases. The FAR may primarily reflect systemic inflammation in chronic and sub-acute conditions, marked by elevated fibrinogen and decreased albumin. However, this trend might differ in AAS, a highly acute condition. Rather than indicating inflammation-driven fibrinogen production, the FAR in AAS may imply a consumptive state, wherein fibrinogen levels decline rapidly while albumin levels remain stable or decrease slightly. Considering the insufficient time for fibrinogen synthesis in response to systemic inflammation, the FAR decreases accordingly.

### Limitations

This study has several limitations that should be considered when interpreting the results. The investigation was conducted at a single center, which may limit the generalizability of our findings to other centers. While we acknowledge the limitations of our single-center design, our study population remains comparable to previous studies. In a large registry study by Evangelista et al. [36], the average age at diagnosis for AAD patients ranged from 63 years to 67 years (our study: 67 years). The proportion of female patients varied from 30% to 35% (our study: 33%), and the mortality rate fell within the reported range of up to 50% (our study: 40%). However, it is important to highlight that we included all cases of AAS, not just AAD.

The retrospective design inherently introduces the potential for selection bias, limits our ability to establish cause-and-effect relationships, and makes it difficult to reliably assess significant comorbidities (e.g., smoking habits, arterial hypertension, and cancer). Additionally, the transfer of patients from the emergency department to specialized units prevented us from systematically tracking further interventions and complications, such as hemorrhagic shock, inflammation, consumptive coagulopathy, transfusions, and sepsis.

Excluding patients with missing fibrinogen and albumin values may have introduced selection bias, as these patients could have had different clinical characteristics or outcomes. However, because laboratory testing was part of routine emergency care, the missing values were likely due to non-systematic factors, which minimized any potential impact on our findings.

One significant limitation of this study is the selective inclusion criteria. We analyzed only patients with AAS confirmed by radiologic imaging, excluding cases where AAS was clinically suspected but not imaged or discovered post mortem. Furthermore, patients lacking FAR values were omitted from the analysis. These exclusions may have introduced selection bias and limited the study’s ability to capture the full spectrum of AAS presentations and outcomes. As a result, our findings may not fully represent all AAS cases, particularly those with atypical presentations or rapid progression. While D-dimer levels could have provided valuable insights, these data were not consistently available in our cohort, narrowing the scope of our analysis. Although our findings highlight the potential clinical relevance of the FAR, its utility in routine clinical practice requires further validation through prospective studies and larger multicenter investigations. This would also address the limited number of variables included in the Cox regression due to the sample size (n = 171) and the number of events (n = 68).

Some patients with AAS may have died before receiving a CT scan. Routine autopsies are not performed at our institution, which is also a well-known issue in other institutions [22]. Thus, we acknowledge that undiagnosed cases may exist, particularly among patients who died suddenly. This limitation is inherent in retrospective studies and may introduce selection bias by underrepresenting the most severe presentations.

## 5. Conclusions

In conclusion, our study demonstrates a significant association between the FAR and 30-day mortality in AAS patients. However, further research is needed to clarify the value of the FAR and establish its practical application in clinical settings. While it cannot replace clinical assessments or imaging results, it could assist in refining risk stratification, improving resource allocation, and enhancing communication with relatives.

## Figures and Tables

**Table 1:** Patient characteristics.

Parameter	Total (n = 171)	Survived (n = 103)	Died Within 30 Days (n = 68)
Age [years], mean (SD)	67 (13.7)	65 (13)	67 (15)
Female, n (%)	56 (33)	30 (29.1)	26 (38.2)
Cardiopulmonary resuscitation (yes), n (%)	35 (20.4)	5 (4.9)	30 (44.1)
Catecholamines (yes), n (%)	100 (58.1)	45 (43.7)	53 (79.4)
Bleeding on admission (yes), n (%)	146 (84.9)	82 (79.6)	64 (94.1)
White blood cell count [G/L, mean (SD)	12.4 (5.4)	12.4 (5.1)	12.5 (5.9)
C-reactive protein [mg/dL], median (IQR)	0.79 (0.23–3.06)	0.89 (0.31–3.74)	0.66 (0.21–1.76)
Platelet count [G/L], mean (SD)	202 (82)	224 (82)	169 (73)
D-Dimer ^1^ [µg/mL], median (IQR)	3.92 (2.25–16.04)	8.1 (18.4)	63.8 (100.2)
Creatinine [mg/dL], median (IQR)	1.2 (0.9–1.6)	1 (0.9–1.4)	1.31 (1.07–2.01)
Bilirubin [mg/dL], median (IQR)	0.44 (0.3–0.7)	0.49 (0.3–0.7)	0.4 (0.21–0.71)
Cholinesterase [kU/L], median (IQR)	5.83 (4.4–7)	6.05 (4.75–7.48)	5.6 (4–6.8)
ASAT [U/L], median (IQR)	26.5 (19–51)	24 (17–35)	38 (23–76)
ALAT [U/L], median (IQR)	23 (14–42)	21 (14–35)	27.5 (16–60)
Gamma-GT [U/L], median (IQR)	29 (18–57)	26 (17–47)	31 (20–64)
Triglyceride [mg/dL], median (IQR)	104.5 (76–146)	100 (72–153)	109 (77–142)
GFR [ml/min/1,73 m^2^], median (IQR)	40.02 (27.6–50.04)	40.71 (30–53.87)	39 (23.5–46.21)

^1^ Available in 45 patients. Abbreviations: ALAT, alanine transaminase; ASAT, aspartate transaminase; gamma-GT, gamma-glutamyltransferase; GFR, glomerular filtration rate

**Table 2:** Fibrinogen, albumin, and fibrinogen-to-albumin ratio (FAR) in the study cohort. Data are presented as mean and standard deviation.

Parameter	Survived	Died	*p*-Value
Fibrinogen [g/L]	3.75/(1.55)	2.76 (1.60)	<0.001
Albumin [g/L]	36.48 (6.49)	32.48 (8.97)	0.001
FAR	10.74 (5.44)	8.89 (4.97)	0.025

**Table 3:** Association of the fibrinogen-to-albumin ratio (FAR) with 30-day mortality. Gender, age, cardiopulmonary resuscitation (CPR), catecholamine administration, and bleeding on admission were added as covariates.

Parameter	HR (95% CI)	*p*-Value
FAR	0.92 (0.87–0.98)	0.010
Sex [male/female]	1.85 (1.12–3.07)	0.017
Age [years]	1.02 (1.00–1.04)	0.116
CPR [yes/no]	6.00 (3.50–10.29)	<0.001
Catecholamine administration [yes/no]	2.02 (1.09–3.73)	0.026
Bleeding on admission [yes/no]	2.47 (0.87–7.00)	0.089
Type of AAS	0.80 (0.58–1.09)	0.159

**Table 4:** The 2018 ISTH DIC Score in our study cohort.

Points	n	%
0	7	18.9
1	12	32.4
2	8	21.6
3	7	18.9
4	2	5.4
6	1	2.7

## Data Availability

The data presented in this study are available on request from the corresponding author.

## Abbreviations

The following abbreviations are used in this manuscript:

AAD	Acute aortic dissection
AAS	Acute aortic syndrome
ADD-RS	Aortic Dissecction Detection Risk Score
ALAT	Alanine transaminase
ASAT	Aspartate transaminase
CI	Confidence Interval
CPR	Cardiopulmonary resuscitation
CRP	C-reactive protein
DIC	Disseminated intravascular coagulation
FAR	Fibrinogen-to-albumin ratio
Gamma-GT	Gamma-glutamyltransferase
GFR	Glomerular filtration rate
HR	Hazard ratio
IMH	Intramural hematoma
ISTH	Internatinoal Society on Thrombosis and Haemostasis
IQR	Interquartile range
PAU	Penetrating aortic ulcer

## References

[1] Daily P.O., Trueblood H.W., Stinson E.B., Wuerflein R.D., Shumway N.E. (1970). Management of Acute Aortic Dissections. Ann. Thorac. Surg..

[2] De Bakey M.E., Henly W.S., Cooley D.A., Morris G.C., Crawford E.S., Beall A.C. (1965). Surgical Management of Dissecting Aneurysms of the Aorta. J. Thorac. Cardiovasc. Surg..

[3] Vilacosta I., San Román J.A. (2001). Acute aortic syndrome. Heart.

[4] Mészáros I., Mórocz J., Szlávi J., Schmidt J., Tornóci L., Nagy L., Szép L. (2000). Epidemiology and clinicopathology of aortic dissection. Chest.

[5] Bossone E., LaBounty T.M., Eagle K.A. (2018). Acute aortic syndromes: Diagnosis and management, an update. Eur. Heart J..

[6] Ferrera C., Vilacosta I., Cabeza B., Cobiella J., Martínez I., Saiz-Pardo Sanz M., Bustos A., Serrano F.J., Maroto L. (2020). Diagnosing Aortic Intramural Hematoma: Current Perspectives. Vasc. Health Risk Manag..

[7] Morello F., Bima P., Castelli M., Capretti E., de Matos Soeiro A., Cipriano A., Costantino G., Vanni S., Leidel B.A., Kaufmann B.A. (2024). Diagnosis of acute aortic syndromes with ultrasound and d-dimer: The Profundus study. Eur. J. Intern. Med..

[8] Cesarman-Maus G., Hajjar K.A. (2005). Molecular mechanisms of fibrinolysis. Br. J. Haematol..

[9] Konstantinides S.V., Meyer G., Becattini C., Bueno H., Geersing G.J., Harjola V.P., Huisman M.V., Humbert M., Jennings C.S., Jiménez D. (2019). 2019 ESC Guidelines for the diagnosis and management of acute pulmonary embolism developed in collaboration with the European Respiratory Society (ERS): The Task Force for the diagnosis and management of acute pulmonary embolism of the European Society of Cardiology (ESC). Eur. Respir. J..

[10] Isselbacher E.M., Preventza O., Hamilton Black J., Augoustides J.G., Beck A.W., Bolen M.A., Braverman A.C., Bray B.E., Brown-Zimmerman M.M., Chen E.P. (2022). 2022 ACC/AHA Guideline for the Diagnosis and Management of Aortic Disease: A Report of the American Heart Association/American College of Cardiology Joint Committee on Clinical Practice Guidelines. Circulation.

[11] Suzuki T., Bossone E., Sawaki D., Jánosi R.A., Erbel R., Eagle K., Nagai R. (2013). Biomarkers of aortic diseases. Am. Heart J..

[12] Suzuki T., Distante A., Zizza A., Trimarchi S., Villani M., Salerno Uriarte J.A., De Luca Tupputi Schinosa L., Renzulli A., Sabino F., Nowak R. (2009). Diagnosis of acute aortic dissection by D-dimer: The International Registry of Acute Aortic Dissection Substudy on Biomarkers (IRAD-Bio) experience. Circulation.

[13] Třeška V., Topolčan O., Pecen L. (2000). Cytokines as plasma markers of abdominal aortic aneurysm. Clin. Chem. Lab. Med..

[14] Erbel R., Aboyans V., Boileau C., Bossone E., Bartolomeo R.D., Eggebrecht H., Evangelista A., Falk V., Frank H., Gaemperli O. (2014). 2014 ESC Guidelines on the diagnosis and treatment of aortic diseases: Document covering acute and chronic aortic diseases of the thoracic and abdominal aorta of the adult. The Task Force for the Diagnosis and Treatment of Aortic Diseases of the European Society of Cardiology (ESC). Eur. Heart J..

[15] Chen C.M., Lu C.F., Liu W.S., Gong Z.H., Wang X.Q., Xu F., Ji J.F., Fang X.X. (2022). Association between fibrinogen/albumin ratio and arterial stiffness in patients with type 2 diabetes: A cross-sectional study. Front. Pharmacol..

[16] Sun D.W., An L., Lv G.Y. (2020). Albumin-fibrinogen ratio and fibrinogen-prealbumin ratio as promising prognostic markers for cancers: An updated meta-analysis. World J. Surg. Oncol..

[17] Li J., Zhou X., Xiang Y., Zhang S., Feng W., Yuan Y., Liu Y., Yin S. (2021). Clinical Significance of Preoperative Fibrinogen to Albumin Ratio in Patients with Glioblastoma: A Singe Center Experience. Cancer Manag. Res..

[18] Ding Y., Xue L. (2022). The potential value of fibrinogen to albumin ratio (FAR) in the assessment of inflammation in spondyloarthritis. BMC Musculoskelet. Disord..

[19] Dai L.L., Chen C., Wu J., Cheng J.F., He F. (2022). The predictive value of fibrinogen-to-albumin ratio in the active, severe active, and poor prognosis of systemic lupus erythematosus: A single-center retrospective study. J. Clin. Lab. Anal..

[20] Zhao Y., Yang J., Ji Y., Wang S., Wang T., Wang F., Tang J. (2019). Usefulness of fibrinogen-to-albumin ratio to predict no-reflow and short-term prognosis in patients with ST-segment elevation myocardial infarction undergoing primary percutaneous coronary intervention. Heart Vessel..

[21] Xiao L., Jia Y., Wang X., Huang H. (2019). The impact of preoperative fibrinogen-albumin ratio on mortality in patients with acute ST-segment elevation myocardial infarction undergoing primary percutaneous coronary intervention. Clin. Chim. Acta.

[22] Wang X., Hu Y., Luan H., Luo C., Kamila K., Zheng T., Tian G. (2023). Predictive impact of fibrinogen-to-albumin ratio (FAR) for left ventricular dysfunction in acute coronary syndrome: A cross-sectional study. Eur. J. Med. Res..

[23] Liu J., Sun L.L., Wang J., Ji G. (2018). The relationship between fibrinogen and in-hospital mortality in patients with type A acute aortic dissection. Am. J. Emerg. Med..

[24] Gao Y., Li D., Cao Y., Zhu X., Zeng Z., Tang L. (2019). Prognostic value of serum albumin for patients with acute aortic dissection: A retrospective cohort study. Medicine.

[25] Grafeneder J., Krychtiuk K.A., Buchtele N., Schoergenhofer C., Gelbenegger G., Lenz M., Wojta J., Heinz G., Huber K., Hengstenberg C. (2020). The ISTH DIC score predicts outcome in non-septic patients admitted to a cardiovascular intensive care unit. Eur. J. Intern. Med..

[26] Suzuki K., Wada H., Imai H., Iba T., Thachil J., Toh C.H. (2018). A re-evaluation of the D-dimer cut-off value for making a diagnosis according to the ISTH overt-DIC diagnostic criteria: Communication from the SSC of the ISTH. J. Thromb. Haemost..

[27] Lee D., Kim Y.W., Kim T.Y., Lee S., Do H.H., Seo J.S., Lee J.H. (2022). Age-Adjusted D-Dimer in Ruling Out Acute Aortic Syndrome. Emerg. Med. Int..

[28] Otani T., Abe T., Ichiba T., Kashiwa K., Naito H. (2023). D-dimer measurement is useful irrespective of time from the onset of acute aortic syndrome symptoms. Am. J. Emerg. Med..

[29] Kotani Y., Toyofuku M., Tamura T., Shimada K., Matsuura Y., Tawa H., Uchikawa M., Higashi S., Fujimoto J., Yagita K. (2017). Validation of the diagnostic utility of D-dimer measurement in patients with acute aortic syndrome. Eur. Heart J. Acute Cardiovasc. Care.

[30] Hazui H., Nishimoto M., Hoshiga M., Negoro N., Muraoka H., Murai M., Ohishi Y., Fukumoto H., Morita H. (2006). Young Adult Patients With Short Dissection Length and Thrombosed False Lumen Without Ulcer-Like Projections are Liable to Have False-Negative Results of D-Dimer Testing for Acute Aortic Dissection Based on a Study of 113 Cases. Circ. J..

[31] Ohlmann P., Faure A., Morel O., Petit H., Kabbaj H., Meyer N., Cheneau E., Jesel L., Epailly E., Desprez D. (2006). Diagnostic and prognostic value of circulating D-Dimers in patients with acute aortic dissection. Crit. Care Med..

[32] Arima D., Suematsu Y., Yamada R., Matsumoto R., Kurahashi K., Nishi S., Yoshimoto A. (2022). Relationship of acute type A aortic dissection and disseminated intravascular coagulation. J. Vasc. Surg..

[33] Guan X.L., Li L., Jiang W.J., Gong M., Li H.Y., Liu Y.Y., Wang X.L., Zhang H.J. (2023). Low preoperative serum fibrinogen level is associated with postoperative acute kidney injury in patients with in acute aortic dissection. J. Cardiothorac. Surg..

[34] Yang S., Xue Y., Liu J., Zhang H., Jiang W. (2020). Is fibrinogen plasma level a risk factor for the first 24-hour death of medically treated acute type A aortic dissection patients?. Ann. Transl. Med..

[35] Rogers A.M., Hermann L.K., Booher A.M., Nienaber C.A., Williams D.M., Kazerooni E.A., Froehlich J.B., O’Gara P.T., Montgomery D.G., Cooper J.V. (2011). Sensitivity of the aortic dissection detection risk score, a novel guideline-based tool for identification of acute aortic dissection at initial presentation: Results from the international registry of acute aortic dissection. Circulation.

[36] Evangelista A., Isselbacher E.M., Bossone E., Gleason T.G., Eusanio M.D., Sechtem U., Ehrlich M.P., Trimarchi S., Braverman A.C., Myrmel T. (2018). Insights From the International Registry of Acute Aortic Dissection: A 20-Year Experience of Collaborative Clinical Research. Circulation.

